# Studying the Gut Microbiome by Amplicon‐Based Metagenomics and Its Relationship with Alzheimer's Disease

**DOI:** 10.1002/alz70856_100663

**Published:** 2025-12-25

**Authors:** Kanthita Worachotsueptrakun

**Affiliations:** ^1^ King Chulalongkorn Memorial Hospital, Pathumwan, Bangkok, Thailand

## Abstract

**Background:**

The imbalance of microbial composition in the gut of elderly individuals can contribute to the development of Alzheimer's disease due to the bidirectional communication between the brain and the gut. Certain groups of gut bacteria can produce metabolites that are toxic to neurons, leading to inflammation and neuronal death in the central nervous system through various pathways. Diet plays a crucial role in influencing the composition of gut bacteria. Consuming prebiotic foods or dietary fibers from vegetables, fruits, and whole grains can stimulate the growth of bacteria that produce short‐chain fatty acids or probiotic microorganisms.

**Method:**

Patients who met the inclusion criteria were recruited for this study. Clinical conditions were assessed, and blood samples were collected to analyze the accumulation of abnormal beta‐amyloid (as the ratio between 42/40), *p*‐Tau 181, brain damage markers (neurofilament light chain [NFL] and glial fibrillary acidic protein [GFAP]), and APOE. Additionally, gut microbiome analysis was performed using amplicon‐based metagenomic methods. The data analysis was conducted in correlation with clinical symptoms.

**Result:**

To confirm the results from the discovery sample, participants were recruited for the study. Gut microbiome compositional analysis was performed on fresh stool samples collected from both the study group (*n* = 40) and age‐ and sex‐matched control participants (*n* = 40). Microbiome features correlated with plasma phosphorylated tau 181 (*p*‐tau181) and plasma neurofilament light chain (NFL), but not with APOE ε3 or neurodegeneration biomarkers, suggesting that changes in the gut microbial community occur early in the disease process. Specific taxa and microbial pathways associated with preclinical Alzheimer's disease were identified.

**Conclusion:**

These beneficial bacteria can produce anti‐inflammatory metabolites and reduce the entry of neurotoxic metabolites into the system. Therefore, understanding the relationship between gut bacteria, prebiotics, and Alzheimer's disease could provide a preventative approach to reduce the risk of Alzheimer's disease caused by microbial imbalance in the elderly.

